# Indirect Time-of-Flight with GHz Correlation Frequency and Integrated SPAD Reaching Sub-100 µm Precision in 0.35 µm CMOS

**DOI:** 10.3390/s23052733

**Published:** 2023-03-02

**Authors:** Michael Hauser, Horst Zimmermann, Michael Hofbauer

**Affiliations:** Institute of Electrodynamics, Microwave and Circuit Engineering TU Wien, 1040 Vienna, Austria

**Keywords:** indirect time-of-flight (iTOF), time-of-flight (TOF), single-photon avalanche diode (SPAD), light detection and ranging (LIDAR), depth sensing

## Abstract

The purpose of this work is to prove the suitability of integrated single-photon avalanche diode (SPAD)-based indirect time-of-flight (iTOF) for sub-100 µm precision depth sensing using a correlation approach with GHz modulation frequencies. For this purpose, a prototype containing a single pixel consisting of an integrated SPAD, quenching circuit, and two independent correlator circuits was fabricated in a 0.35 µm CMOS process and characterized. It achieved a precision of 70 µm and a nonlinearity of less than 200 µm at a received signal power of less than 100 pW. Sub-mm precision was achieved with a signal power of less than 200 fW. These results and the simplicity of our correlation approach underline the great potential of SPAD-based iTOF for future depth sensing applications.

## 1. Introduction

Optical ranging methods are increasingly gaining in importance, especially for 3D sensors in the form of light detection and ranging (LIDAR) systems and time-of-flight (TOF) pixel sensors. TOF sensors stand out due to their simplicity, allowing for cheap manufacturing and their use in mass products, such as mobile phones, etc. Nevertheless, available sensors are either limited in precision or sensitivity.

There are two main approaches for TOF sensing. The most widespread approach is direct time-of-flight (dTOF). Here, measuring the time delay between transmission and reception of a light pulse, i.e., the time-of-flight, allows for the calculation of the distance to the reflecting object. For assuring a sufficient range and distance resolution, usually single-photon avalanche diodes (SPADs) are used as photodetectors. Due to the high speed of light, for achieving sub-mm accuracies, timing accuracies must be better than 6.6 ps, which is hard to accomplish with the used time-to-digital converters (TDCs). In [1], sub-mm depth precision was achieved with an integrated dTOF system by applying averaging over 1000 successive measurements. Nevertheless, depth precisions of dTOF systems are typically in the order of millimeters [2,3,4,5,6].

In contrast to direct time-of-flight, there is also indirect time-of-flight (iTOF), where, instead of a direct time measurement, a phase measurement is performed. There is a variety of approaches for implementing this phase measurement. iTOF systems using pinned photodiodes (PPDs) achieved sub-mm [7,8] or even sub-100 µm [9] depth precisions, but their range is limited. SPADs allow much higher sensitivities than PPDs. Nevertheless, to the best of our knowledge, there is no work achieving sub-100 µm precision with SPADs. In [10], a depth precision of 7.8 mm was achieved with a SPAD-based time-gated TOF approach. The best depth precision of a SPAD-based iTOF sensor of 1 mm was achieved in [11], which uses the same phase measurement approach as this work, but performs the proposed correlation digitally and with a lower modulation frequency. Simulation results of a similar design as this work were published in [12]. However, due to limited simulation time, only mm-accuracies were simulated.

There are also more complex forms of TOF, for example, nonlinear optical gating (e.g., [13]). Here, the received signal photons are optically gated with a pump pulse in a nonlinear process. Using short pump pulses would potentially allow for precisions in the order of micrometers. Nevertheless, such systems are cost intensive and relatively complex.

The purpose of this work is proving that our analog single-photon correlation approach in combination with a high modulation frequency allows achieving sub-100 µm precision iTOF with integrated SPADs. The achieved precision, sensitivity, and simplicity of this concept enables a wide range of new applications for TOF sensors. A prototype was manufactured in a 0.35 µm CMOS process, and depth precision, linearity, background light suppression, and SPAD excess voltage dependence were characterized.

## 2. iTOF with Analog Single-Photon Correlation

**Figure 1:**
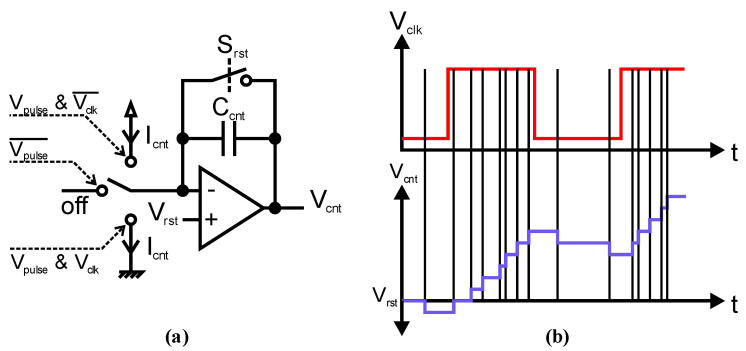
(**a**) Schematic of an integrator-based analog correlator circuit. Logic combinations for the different switch positions are depicted with dashed lines; (**b**) integration of photon pulses and *V_clk_* over time *t* with photon pulses marked as vertical black lines.

Our iTOF approach is based on a phase measurement by correlation of SPAD-detected photons with a phase-shiftable reference clock *V_clk_*. Figure 1a shows the schematic of the integrator-based analog correlator proposed in this work. The integrator with a capacitor *C_cnt_* forms an analog counter. A preceding SPAD quenching circuit generates a voltage pulse *V_pulse_* for every detected photon. If there is a pulse, by setting the appropriate switch position, a decision logic decides if current *I_cnt_* is charging or discharging *C_cnt_* for the pulse time *T_pulse_*, and hence if the analog counter is counting upwards or downwards. The voltage step *V_step_* for a single count is given by:

(1) $$V_{step}=\frac{I_{cnt}*T_{pulse}}{C_{cnt}},$$

and the counting direction is set by the actual *V_clk_* logic level, i.e., it counts up if there is a photon pulse and *V_clk_* is high or down if low. If there is no photon pulse, the integrator input node is kept floating, and hence *V_cnt_* is kept constant. The different logic combinations for the switch are depicted with dashed lines. In Figure 1b, *V_cnt_* is plotted over time *t* for a given *V_clk_* and photon pulses. The reset switch is closed and opened again before the start of this integration in order to reset *V_cnt_* to the reset voltage *V_rst_*. In the illustrated example, *V_clk_* and photon pulses are in phase, which causes *V_cnt_* to increase over time.

**Figure 2:**
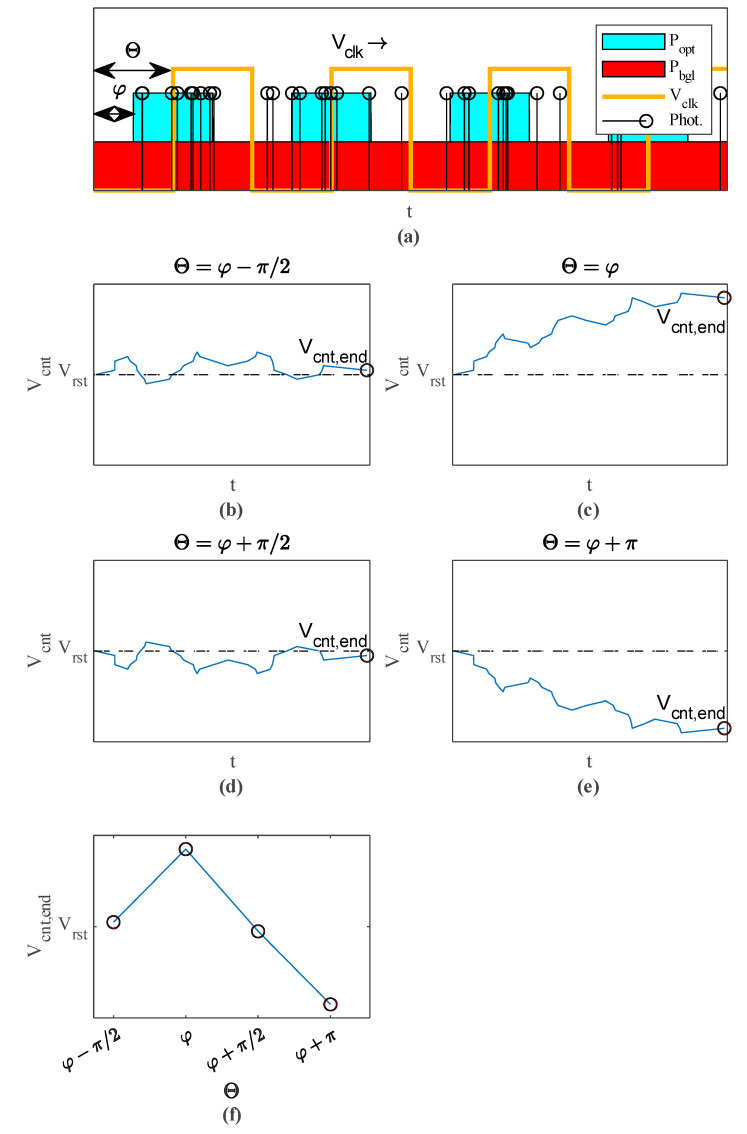
Basic principle of iTOF measurement with our correlation approach: (**a**) probabilities for receiving a signal photon pulse *P_opt_* or a background light photon pulse *P_bgl_* with shown photon pulses and *V_clk_* over time *t*; (**b**–**e**) integration of these photons for four different phase steps *Θ* with plotted *V_cnt_* over *t*; (**f**) *V_cnt_* end values merged to a correlation triangle.

For measurements with a modulation frequency of 1 GHz, the SPAD deadtime of about 10 ns is much longer than the modulation period of 1 ns. Hence, also for a high photon rate, an integration time of at least several hundred modulation periods is necessary to achieve reasonable results. As such a measurement would be hard to plot, for the examples in Figure 1 and Figure 2, a rather small modulation frequency of about 1 MHz was chosen for a better understanding.

Figure 2 shows a complete iTOF measurement example with four different simultaneously measured *V_clk_* phase steps *Θ*. In Figure 2a, the received light intensity, which consists of a modulated signal part *P_opt_* and a background light part *P_bgl_* is plotted over time. The probability for a photon pulse at time *t* is directly proportional to the combined light intensities. *P_opt_* is phase-shifted by *φ,* which is proportional to the measured distance.

Figure 2b–e shows the evolution of *V_cnt_* when the pulses in Figure 2a are integrated with four π/2-spaced *V_clk_* phase shifts *Θ*. Photon pulses caused by background light or SPAD dark counts are suppressed over a long enough integration time *T_int_*, as they are evenly distributed over all *V_clk_* high and low states. For low modulation frequencies, these pulses cause a continuous up and down counting depending to the current *V_clk_* level. This effect is visible as a superimposed noisy triangular waveform in Figure 2b–e and disappears for higher modulation frequencies. Background photons and SPAD dark counts cause, on average, as many up as down counts and do not affect *V_cnt_* for a long enough *T_int_* as they are averaged out. For sufficiently long *T_int_*, only signal photons contribute to the integration result. After the integration time *T_int_* the *V_cnt_* end values *V_cnt,end_* are read out with an analog-to-digital converter (ADC). Then, by plotting *V_cnt,end_* over *Θ* a correlation triangle is formed (Figure 2f), as the cross correlation of two square functions gives a triangle. This triangle peaks at *Θ* = *φ*, hence measuring the phase of this triangle allows the determination of *φ*. In this work, this phase measurement is done by performing an FFT and extracting the phase of the fundamental. The relative distance *d* measured in an iTOF measurement is proportional to the time delay *Δt*, the time-of-flight:

(2) $$d=\frac{c*\Delta t}{2}$$

The factor of ½ comes from the fact that the received signal light travels the distance *d* two times, from the light source to the target and back to the receiver.

As the measured phase shift *φ* is proportional to *Δt* in a certain modulation period, *d* can be calculated with:

(3) $$d=\frac{c*\varphi }{4*\pi *f_{mod}}N_{period},$$

where *N_period_* is an integer indicating in which period *φ* lies. As apparent, with a given angular measurement precision of *φ*, the precision of distance *d* can be improved by increasing the modulation frequency *f_mod_*. Hence, for achieving a good precision, a high *f_mod_* is desirable. In this work, a modulation frequency of 1 GHz is used. On the other hand, a higher *f_mod_* decreases the unambiguous range as there is a phase wrap when *φ* exceeds 2π and, without prior knowledge, it is not possible to determine *N_period_* anymore. For 1 GHz, this unambiguous range is 15 cm. Nevertheless, there are solutions to this problem, which are discussed in Section 6.

## 3. Circuit Design

**Figure 3:**
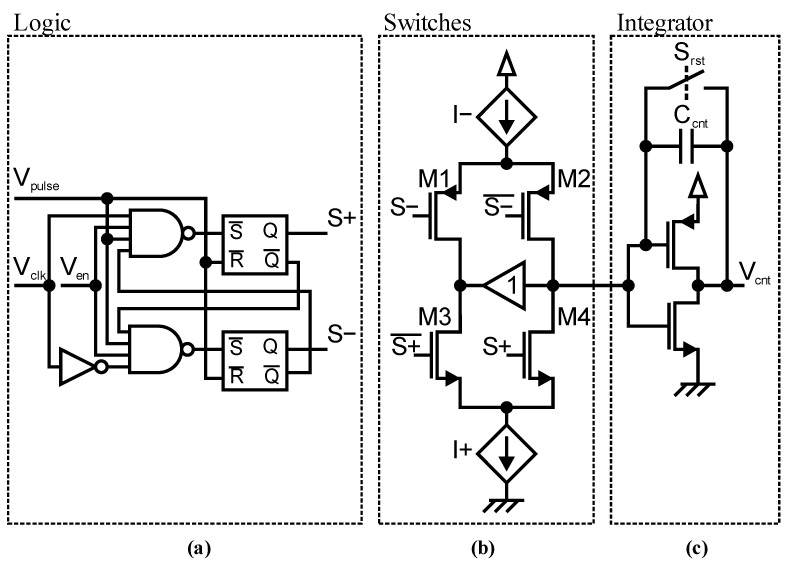
Analog correlator circuit consisting of (**a**) decision logic; (**b**) current steering switches; (**c**) integrator with reset.

The analog correlator was implemented as shown in Figure 3. Figure 3a shows the logic block, which makes the up/down counting decision and generates the control signals *S+* and *S−* for the following analog counter. An additional enable control signal *V_en_* allows for the accurate definition of the integration time *T_int_* and to disable the integration during readout. The complete decision logic consists of just six standard logic NANDs and, due to the edge-triggered operation, high modulation frequencies are possible. The integrator with a simple CMOS inverter amplifier and capacitor *C_cnt_* in Figure 2c together with the two current-steering switch pairs (Figure 2b: M1/M2, M3/M4) for either up or down counting forms the analog counter. If there is no counting (*S+* and *S−* are low), the switches at the integrator input node (M2, M4) are open, while the other switch of each pair (M1, M3) is closed in order to keep the current sources on. Hence, no current is charging *C_cnt_*. If there is, for example, an up count, M3 is opened and M4 is closed to charge *C_cnt_* with current *I+* for the pulse length *T_pulse_*. The voltage step *V_step_* for a count can be adjusted with *T_pulse_*, which is adjustable with an external voltage in the quenching circuit and by adjusting the voltage-controlled current sources *I+* and *I−*, which are also controllable with external voltages. As the maximum counter value, i.e., the dynamic range of the counter, is directly linked with the possible iTOF measurement precision, small *V_step_* sizes are necessary. This is achieved with a short *T_pulse_* (several nanoseconds), small *I+*/*I−* (~100 nA–10 µA), and a large *C_cnt_* (18 pF). The absolute maximum operating range for *V_cnt_* is limited to a range between 0.6 V and 2.9 V due to the limited operating voltage range of the following analog buffer, which buffers *V_cnt_* to the external ADC. For this buffer a simple CMOS operational transconductance amplifier (OTA) is used. The voltage of the integrator input node is copied with an analog buffer to the node between M1 and M3 in order to keep the potential for the current sources constant while switching. For this analog buffer, the same simple CMOS OTA design is used. A transmission gate is used as reset switch, which is controlled with an external reset signal *S_rst_*. With a supply voltage of 3.3 V, the reset voltage *V_rst_* is about 1.6 V.

A complete correlator consists of the integrator with *C_cnt_*, the counter switches, decision logic, voltage-controlled current sources *I+* and *I−*, and a CMOS-OTA to buffer *V_cnt_* to the external ADC. Voltage steps for up and down counting have to be balanced with *I+* and *I−* to compensate switching asymmetries. For this purpose, a calibration circuit was added to give the possibility for a continuous measurement of the *V_step_* values for up and down counting. This calibration circuit is basically a copy of the analog counter using copies of *I+* and *I−* currents, but with the difference of using the *S+* control signal for both current-steering switch pairs; hence, it simultaneously counts upwards and downwards. If up and down counting voltage steps are balanced, the integrator output voltage of the calibration circuit stays constant. If it is changing, either *I+* or *I−* can be adjusted until a sufficient balancing is achieved. For this calibration circuit, a much smaller *C_cnt_* is sufficient. Nevertheless, as explained in Section 4, this extra circuit was not necessary and was not used for iTOF measurements.

**Figure 4:**
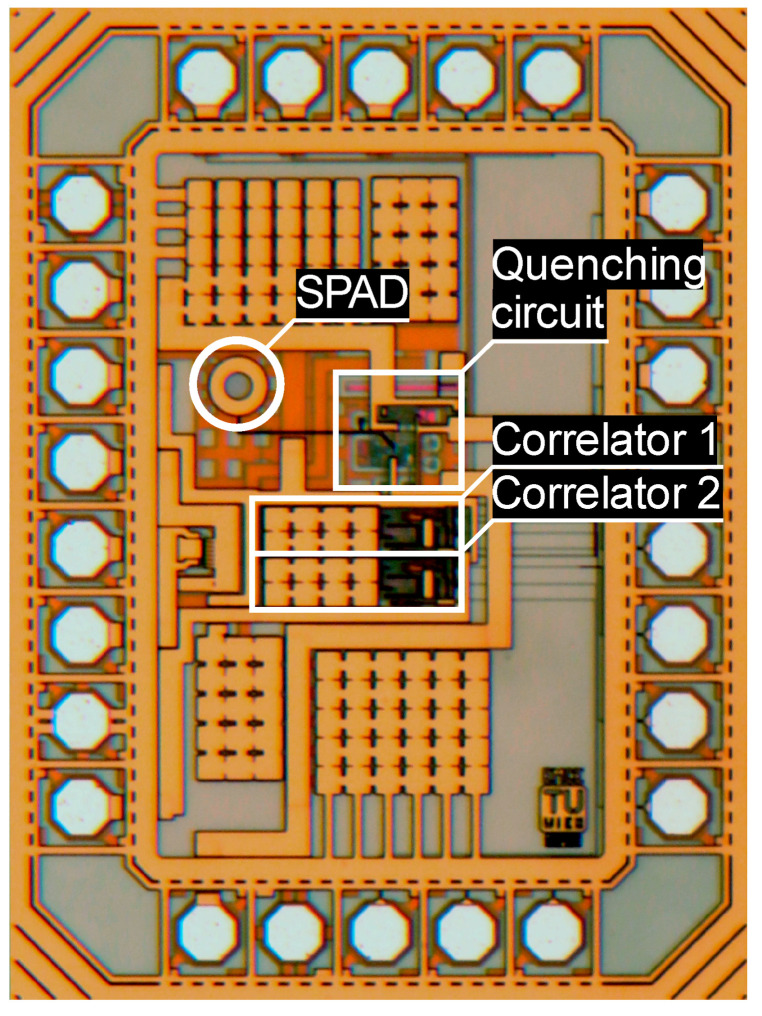
Photomicrograph of the fabricated prototype, with marked SPAD, quenching circuit, and correlators.

Figure 4 shows a photomicrograph of the produced prototype. It consists of an integrated SPAD with quenching circuit for which reliable, already published designs [14,15] with slight improvements of the SPAD were used. The SPAD has an active area with a diameter of 38 µm and a breakdown voltage of 35.9 V. The quenching circuit is able to provide a SPAD excess voltage *V_ex_* of up to 6.6 V and measures 130 µm × 130 µm. A single correlator has a size of 270 µm × 70 µm, whereof more than half of the area is used for the 18 pF metal–insulator–metal (MIM) type counting capacitor *C_cnt_*. This is clearly visible in Figure 4 (left part of the correlator areas). The active part of the correlator measures 110 µm × 70 µm, whereof the decision logic occupies an area of 30 µm × 25 µm. Two independent correlators sharing the photon pulse output of the quenching circuit, but with independent *V_clk_* inputs, are implemented on the chip. Hence, two phase steps *Θ* could be measured simultaneously for reducing measurement time. The whole chip has a size of 1040 µm × 1400 µm, but as the minimum size is given by the number of pads, only a small part contains circuitry. Empty space was filled with decoupling capacitors.

## 4. Measurement Setup

**Figure 5:**
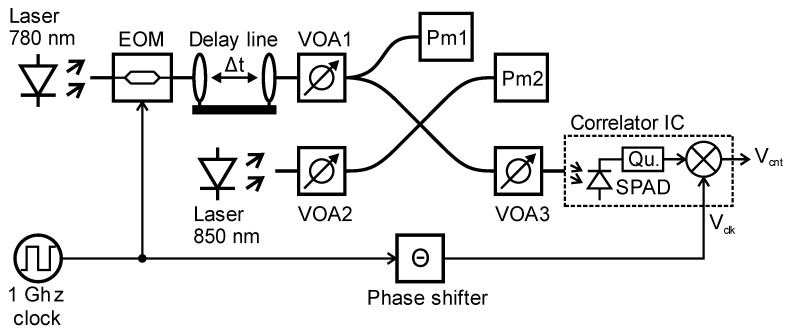
Measurement setup.

Figure 5 shows the used measurement setup. After a Thorlabs (Newton, NJ, USA) LPS-PM785-FC laser with a wavelength of 783.4 nm, the light is amplitude modulated with the 1 GHz clock using an iXblue (Besancon, France) NIR-MX800-LN electro-optic modulator (EOM). The following OZ Optics (Ottawa, ON, Canada) ODL-300 adjustable fiber optic delay line allows for the accurate sweeping of the measured delay over a range from 0 ps to 333 ps. The variable optical attenuator VOA1 allows for the sweeping of the optical signal power over a range of 60 dB. After VOA1, a splitter divides the signal for measuring the signal power with power meter Pm1. Background light is generated with an 850 nm laser and adjusted in power with VOA2. For this task, the wavelength of the light source is not critical, as the purpose of this laser is only to trigger SPAD counts, which are not correlated to the signal light as would background light from other light sources or the sun. Signal and background light is combined with a 2 × 2 combiner, where one output is used for power measurement of the combined light intensities, which is performed with a Thorlabs PM100USB power meter (Pm2). The second output of the 2 × 2 combiner is further attenuated with VOA3 and then fed into the integrated SPAD of the proposed single-photon correlator. The coupling ratio of the 2 × 2 combiner and the attenuation of the fiber and VOA3 are carefully measured for both used wavelengths in order to be able to accurately determine the light intensity reaching the SPAD. The quenching circuit generates a pulse for every detected photon. The phase steps *Θ* of the reference clock signal *V_clk_* are generated with a Microchip (Chandler, AZ, USA) SY89295UTG adjustable phase shifter. A National Instruments (Austin, TX, USA) CompactRIO system generates the correlator control signals, controls the phase shifter, and reads the correlation result *V_cnt,end_* with an NI-9205 16-bit voltage input module. The complete measurement system is controlled by PC and all measurements run fully automated with LabVIEW (15.0). For flexibility reasons, data processing and analysis is done after the measurement in MATLAB (R2022b).

As already explained in Section 3, in order to prevent asymmetries between up and down counting voltage steps, a balancing by either adjusting *I+* or *I−* is necessary. One possibility to measure this asymmetry is using the integrated calibration circuit. Either *I+* or *I−* is adjusted until the output voltage of the calibration circuit stays constant; hence, up and down counting voltage steps are identical. Another possibility, which was used for this work, is to measure the asymmetry with the correlator circuit itself. This is done by using either background light photons or SPAD dark counts without modulated signal light. Then, an integration with applied *V_clk_* is performed with the correlator. As the number of photons or dark counts while *V_clk_* high and low levels is now identical for a sufficiently long integration time, only an asymmetry between up and down counting voltage steps will cause a change of *V_cnt_*. As *V_cnt_* changes according to the direction of the asymmetry, *I+* or *I−* can be adjusted until this asymmetry is sufficiently suppressed. As no voltage step drifting was observed over several weeks of measurement, it seems to be sufficient to perform this balancing once.

For a distance measurement, consecutive integrations for the different *V_clk_* phases *Θ* with integration time *T_int_* are performed. After every integration, *V_cnt,end_* is read out and a correlation triangle is formed. Then, *φ* is determined with an FFT and *d* is calculated with Equation (3).

## 5. Measurements and Results

The measurement setup explained in Section 4 allows for a full characterization of the proposed iTOF system for distance measurement.

### 5.1. Precision at Optical Signal Power

**Figure 6:**
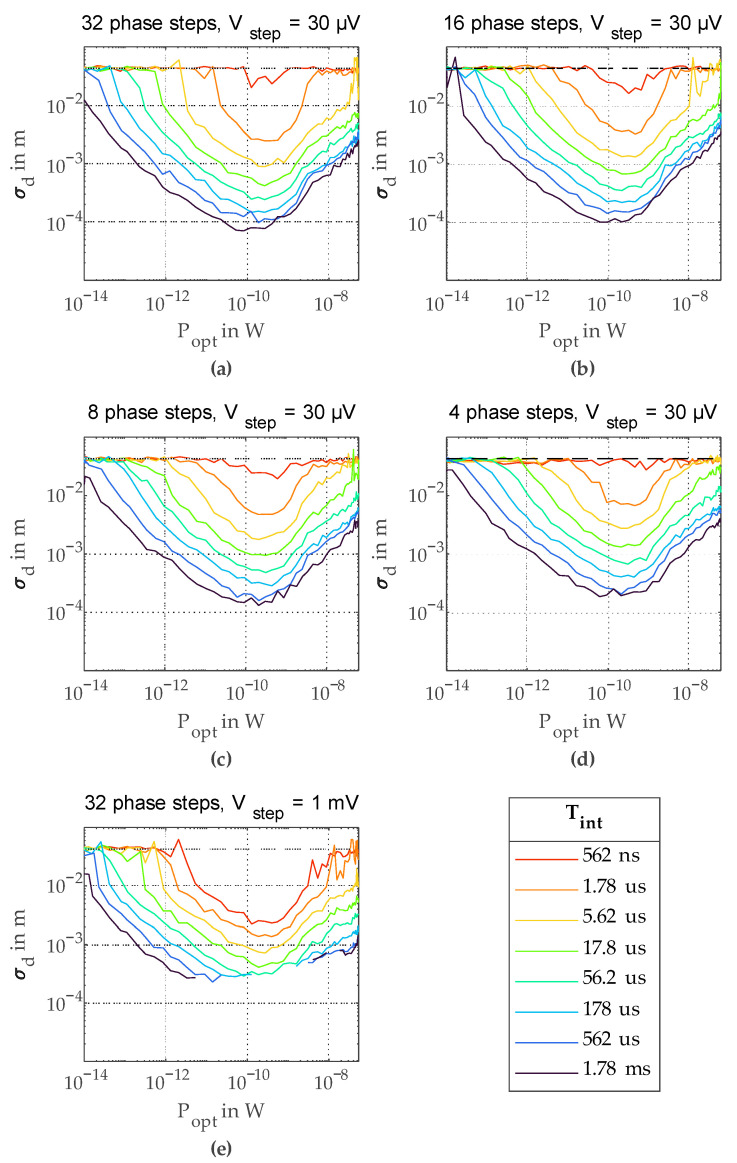
Standard deviation *σ_d_* over *P_opt_* for different *T_int_*.: (**a**) with 32 phase steps, *V_step_* = 30 µV; (**b**) with 16 phase steps, *V_step_* = 30 µV; (**c**) with 8 phase steps, *V_step_* = 30 µV; (**d**) with 4 phase steps, *V_step_* = 30 µV; (**e**) with 32 phase steps, *V_step_* = 1 mV.

One of the main parameters of an optical distance measurement system is the achieved depth precision, and hence, the deviation of consecutive distance measurements with a fixed measurement distance. As measured distance values are usually Gaussian distributed, a very good measure for the depth precision is the standard deviation of these values. For achieving a good standard deviation *σ_d_*, a sufficient number of samples is necessary. In this work, 100 consecutive distance measurements were taken for every *σ_d_* value. A first informative measurement is measuring *σ_d_* for a series of optical signal powers *P_opt_* and different integration times *T_int_*. The results of this measurement for a varying number of phase steps are shown in Figure 6. The dashed horizontal lines mark the worst possible *σ_d_* value, i.e., the *σ_d_* value for distance values, which are completely evenly distributed over the whole unambiguous range. Hence, if the calculated *σ_d_* reaches this line, the measured values do not contain any useful distance information anymore. In Figure 6, one can see a clear improvement of *σ_d_* with increasing integration time *T_int_*. This is due to the increasing number of photons contributing to the measurement and a growing amplitude of the correlation triangle. There is an optimum value of *P_opt_*, where the best *σ_d_* is achieved. For higher *P_opt_* values, *σ_d_* gets worse again, mainly due to saturation of the SPAD. The best precision with a *σ_d_* of 70 µm was achieved with 32 consecutive phase steps and a *T_int_* per phase step of 1.78 ms at a *P_opt_* of 80 pW (Figure 6a). Figure 6b–d shows *σ_d_* measurements with a decreasing number of phase steps. Even with the minimum number of four phase steps, a precision of 186 µm was achieved.

In Figure 6e, the counting voltage step *V_step_* was increased by approximately a factor of 30 from 30 µV to 1 mV by increasing *I+* and *I−*. This improved the performance for shorter *T_int_* in comparison to the measurements with smaller *V_step_* in Figure 6a considerably. This is due to the fact that the amplitude of the correlation triangle gets very small for short *T_int_*. Hence, electronic noise while reading out *V_cnt,end_* starts to play a role. A significant part of this noise originates from long, unshielded lines between the chip-printed circuit board (PCB) and the NI-9205 voltage input module used for *V_cnt,end_* readout. This influence can be reduced considerably in the future by bringing the ADC closer to the output of the analog correlator. For the measurements with the increased *V_step_*, there is no visible influence of electronic noise. On the other hand, as the operating area for *V_cnt_* is limited, increasing *V_step_* decreases the dynamic range of the analog counter. This limited dynamic range causes saturation of the correlation triangle for higher *P_opt_* and *T_int_*, which is clearly visible in Figure 6e, where *σ_d_* values with saturation were removed as no reasonable calculation of *σ_d_* was possible anymore. Depending on the application, especially on the used *T_int_* and the received *P_opt_*, different *V_step_* values will give the best results.

**Figure 7:**
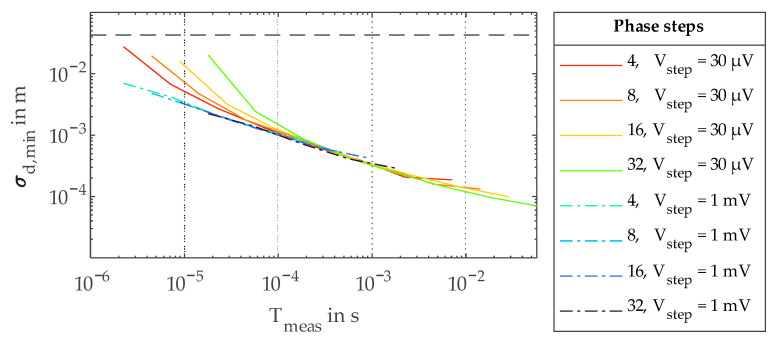
Minimum standard deviation *σ_d,min_* over total measurement time *T_meas_*.

In Figure 7, the minimum *σ_d_* values *σ_d,min_* for all measured combinations of number of phase steps and *V_step_* are plotted over the total measurement time for a single distance measurement *T_meas_*. As in this work, the different phase step measurements were performed consecutively, and *T_meas_* is given by the number of phase steps times *T_int_*. One can clearly see the logarithmic behavior of *σ_d,min_* for increasing *T_meas_*. For smaller *T_meas_* values, the curves with the smaller *V_step_* value deviate from the ideal line due to the above explained electronic noise problem. Nevertheless, with the larger *V_step_*, this logarithmic behavior is extended down to microseconds of measurement time. For longer *T_meas_*, *σ_d_* is limited by the dynamic range of the analog counter.

### 5.2. Nonlinearity at Optical Signal Power

**Figure 8:**
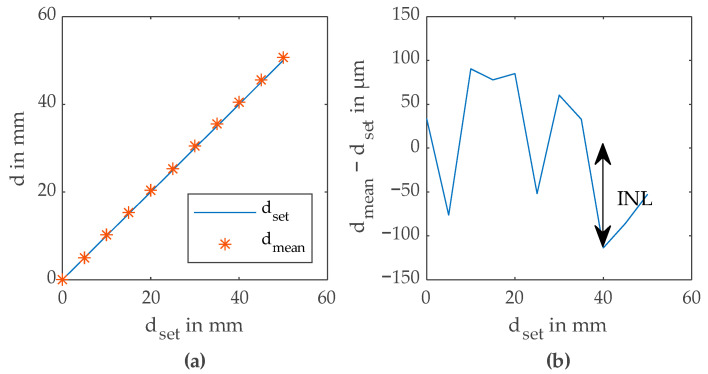
(**a**) Distance *d_set_* and averaged measured distance *d_mean_* over *d_set_*; (**b**) measurement of *INL*.

**Figure 9:**
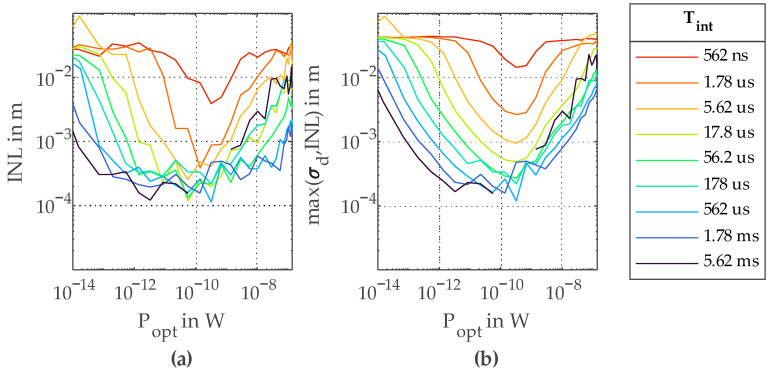
(**a**) Integral nonlinearity *INL* over *P_opt_* for different *T_int_*; (**b**) maximum of *INL* and *σ_d_* over *P_opt_* for different *T_int_*.

For the linearity measurements, the adjustable delay line was used. To measure the integral nonlinearity *INL*, the delay *Δt* set by the delay line was swept between 0 ps and 333 ps. With Equation (2), this gives a distance sweep of 50 mm. For all 11 distance steps of a single sweep, 100 consecutive measurements were performed in order to get the average distance value *d_mean_* and the standard deviation *σ_d_*. The offset of *d_mean_* was calculated using a least squares fit and subtracted from all *d_mean_* values. Figure 8a shows the distance *d_set_* and the measured distance *d_mean_* for one distance sweep. The maximum deviation of all 11 measured *d_mean_* from *d_set_* is used as *INL*, as visible in Figure 8b. This measurement was repeated for several *P_opt_* and *T_int_*. As visible in Figure 9a, the best *INL* achieved for these measurements is 113 µm. For very long *T_int_*, the measured *INL* is worsening again due to saturation of the correlator. In Figure 9b, the maximum of *σ_d_* and *INL* is plotted. This plot gives an estimate of the expectable total accuracy of a single measurement.

### 5.3. Background Light Suppression

**Figure 10:**
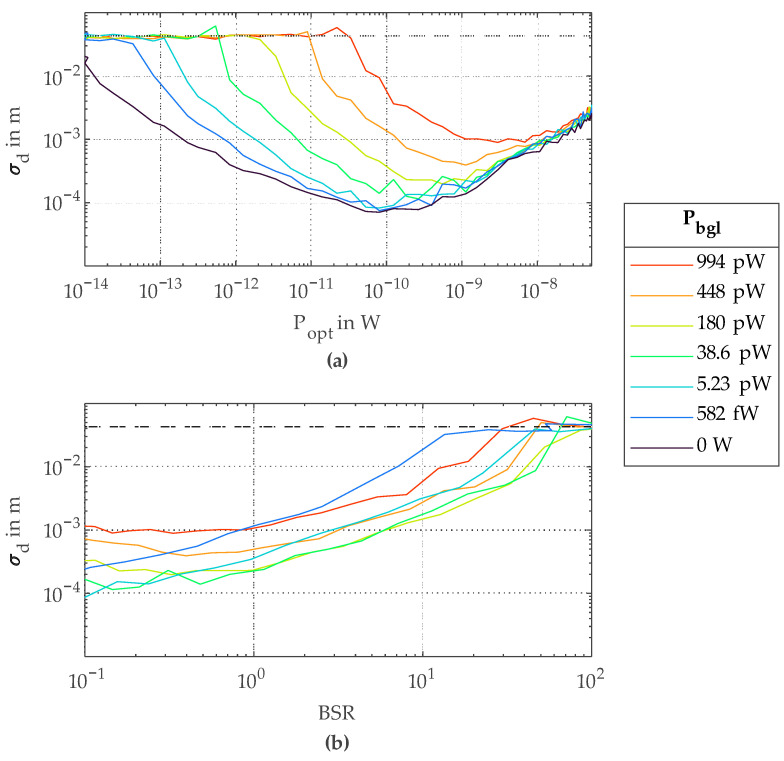
Measurements with added background light using 32 phase steps and a *T_int_* of 1.78 ms: (**a**) standard deviation *σ_d_* over *P_opt_* for different *P_bgl_*; (**b**) standard deviation *σ_d_* over *BSR* for different *P_bgl_*.

Immunity against background light is of great importance for many realistic measurement scenarios. Due to the correlation approach used in this work, background light photons are suppressed as they are evenly distributed over all up and down counting phases given by *V_clk_* and are averaged out. Nevertheless, these photons do have an impact on the measurement result, as they can saturate the SPAD and fewer signal photons are detected. Figure 10 shows the achieved results with added background light. The background light power *P_bgl_* generated with the 850 nm laser (Figure 5) was swept with VOA2. The photon detection probability *PDP* of the SPAD at 850 nm is about 15 % smaller than at 780 nm [14]. On the other hand, the photon energy at 850 nm is about 10 % smaller compared to 780 nm, which leads to comparable photon rates at both wavelengths for the same optical power. In Figure 10a, one can see how the achieved *σ_d_* worsens with increasing *P_bgl_*. With increasing *P_bgl_*, a higher *P_opt_* is necessary to achieve acceptable *σ_d_* values. Furthermore, the dynamic range regarding *P_opt_*, for which a specific *σ_d_* is achieved, is decreased. The reasons for this decrease are additional noise due to background photons and a decrease of received signal photons if background photons saturate the SPAD. Figure 10b shows *σ_d_* plotted over the background-to-signal-ratio *BSR*. In an application, the influence of background light can be greatly reduced by using narrow laser line filters in front of the detector.

### 5.4. Dependence on SPAD Excess Voltage V_ex_

**Figure 11:**
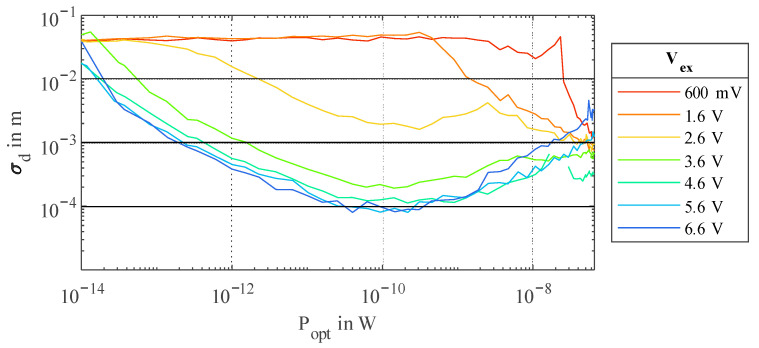
Standard deviation *σ_d_* over *P_opt_* for different *V_ex_* with 32 phase steps and a *T_int_* of 1.78 ms.

As the *PDP* of the integrated SPAD decreases with a decreasing SPAD excess voltage *V_ex_*, less photons are detected. On the other hand, afterpulsing of the SPAD, caused by trapped charge carriers left from previous avalanches, is also decreased. Afterpulsing could theoretically worsen the measurement results. Nevertheless, as visible in Figure 11, the effect due to decreasing *PDP* seems to exceed the effect of decreased afterpulsing by far, and hence, a high *V_ex_* should be chosen.

## 6. Discussion

The purpose of this work is to prove the ability of SPAD-based iTOF with high frequency correlation to achieve sub-100 µm precisions. These good precision results were achieved by the use of a modulation frequency *f_mod_* of 1 GHz. Equation (3) shows the relationship between the measured distance and *f_mod_*. The high *f_mod_* is possible due to the more digital operation of SPADs. The decision between up and down counting can be performed with a fast digital circuit. The achieved precision and linearity in combination with the achieved sensitivity can open up new high-precision measurement applications for TOF sensors, which, so far, depend on more complex interferometric sensors.

Table 1 shows a comparison of high-precision dTOF and iTOF results. To the best of our knowledge, there is no previous work achieving sub-100 µm precisions with SPAD-based integrated TOF. In comparison with integrated dTOF systems, which can achieve precisions in the sub-mm and mm-range, this is an improvement of one order of magnitude. Sub-100 µm precisions were already achieved with PPD-based iTOF. Nonetheless, the SPAD allows much better sensitivities, i.e., requires considerably less light to achieve the same precision.

In contrast to [11], where digital counters were used, in this work, we use a faster edge-triggered decision circuit in combination with an analog counter. The use of an analog counter promises a higher pixel number especially for large feature size technologies due to smaller area and power consumption. Furthermore, as the analog counter allows for an adjustment of the dynamic range by adjusting *V_step_*, it is very flexible in terms of possible integration time in contrast to a digital counter with a strictly limited counting range. Nevertheless, for applications with the need of very long integration times, digital correlators might be favorable as their dynamic range is not limited by electronic noise.

A single pixel prototype was manufactured with an integrated SPAD and quenching circuit. For testing purposes, a quenching circuit with an already proven design [14,15] and wide tunability was chosen. It was not optimized for size and consumes a fairly large area (130 µm × 130 µm). Nevertheless, by using area-optimized quenching circuits and smaller feature size technologies this area consumption could be greatly reduced. The same is true for the correlator circuit, which was optimized for flexibility in order to be able to test all parameters and to perform long integrations with many photons. As more than half of the correlator size of 270 µm × 70 µm is used for the counting capacitor *C_cnt_*, reducing this capacitor size, e.g., by using MOS capacitors or by using smaller application-optimized capacitor values, could save a lot of space. Even a small capacitance of several hundred femtofarads would allow sub-mm precisions. Additionally, a further reduction of *C_cnt_* might be possible by further reducing *I+*/*I−*. Nevertheless, an eye must be kept on additional noise due to small currents. In a similar way, shorter *T_pulse_* values would also allow for the reduction of *C_cnt_*. In this work, *T_pulse_* cannot be reduced to values smaller than 4 ns. As the added calibration circuit, which occupies around 30 % of the active correlator area, turned out not to be necessary, it would be possible to remove it. The active area, with SPAD, quenching circuit, and two correlators, measures about 300 × 300 µm^2^. Therefore, with the actual prototype in 0.35 µm CMOS, a multi-pixel design would be limited to a low number of pixels for a reasonable cost. Implementing a pixel design by taking into account all the previously mentioned considerations would lead to a drastic shrinking of the occupied area per pixel. Hence, multi-pixel TOF imagers are possible, even with the actual fairly large feature size technology. Switching to smaller feature size technologies would lead to a further pixel size reduction.

Increasing the dynamic range of the analog counter, for example by increasing *C_cnt_* or further decreasing *V_step_*, would allow for longer integration times. Extrapolating the logarithmic relationship in Figure 7 allows an estimation of achievable measurement precisions. Achieving precisions of 10 µm or even better might be possible with measurement times of less than a second. Nevertheless, it would be hard to combine such precisions with a high pixel number.

As in this work, the different phase steps were measured consecutively, the total measurement time could be drastically reduced by adding parallel correlators for every phase step. Hence, a simultaneous measurement of all phase steps would be possible.

One major drawback of the actual measurements with a simple square wave modulation is the very limited unambiguous range, which for the used *f_mod_* of 1 GHz is just 15 cm. One solution for this problem would be using two modulation frequencies [16]. Another solution is using orthogonal codes for the signal modulation [17,18,19]. This would bring the additional feature of high immunity to interferers. By using orthogonal coding, the unambiguous range could be simply adjusted with the code length, hence allowing for the combination of a high frequency measurement with a high unambiguous range. In this case, the maximum measurement range would be limited by the sensitivity of the SPAD. Depending on the optics and background light conditions, with an eye-safe laser, a measurement range of at least several 10s of meters should be easily possible. Nevertheless, for applications with high background light levels, narrow laser line filters might be necessary to prevent saturation of the SPAD.

## 7. Conclusions

Our correlation-based indirect time-of-flight (iTOF) approach in combination with a high modulation frequency of 1 GHz allowed us to achieve outstanding results for integrated SPAD-based iTOF with regard to precision, linearity, and sensitivity. A prototype was implemented in a 0.35 µm process and characterized. It consists of a single iTOF pixel containing an integrated SPAD, quenching circuit, and two independent correlator circuits. With this prototype, a distance precision of 70 µm and a nonlinearity of less than 200 µm was achieved over a range of 50 mm. These results were achieved with optical signal powers of less than 100 pW. Sub-mm precision was achieved with signal powers of less than 200 fW.

## Figures and Tables

**Table 1:** Comparison of integrated dTOF and iTOF results.

	This Work	[1]	[2]	[3]	[9]	[11]
Type	iTOF	dTOF	dTOF	dTOF	iTOF	iTOF
Detector	SPAD	SPAD	SPAD	SPAD	PPD	SPAD
Year	2023	2018	2019	2020	2019	2022
Techn.	0.35 µm	0.35 µm	0.18 µm	0.15 µm	0.11 µm	0.35 µm
Pixels	1	9 × 9	252 × 144	50 × 40	192 × 4	1
Chip size	1 × 1.4 mm^2^ ^(1)^	2.5 × 4 mm^2^	21 × 10 mm^2^	3.3 × 2.9 mm^2^	9 × 7 mm^2^	1.4 × 1.4 mm^2^
*σ_d_*/*RMS*	70 µm	<1 mm ^(2)^	1.4 mm	<1.6 mm	64 µm	1 mm
*@ P_opt_*	80 pW	25 pW ^(3)^	-	-	-	80 pW
@ *T_meas_*	57 ms	10 ms ^(4)^	33 ms ^(5)^	1 ms ^(5)^	40 ms	8 s
*INL*	<0.2 mm	±0.5 mm	8.8 mm	<19 mm	0.25 mm	-
Range	150 mm ^(6)^	34 m	50 m ^(7)^	7.5 m	25 mm	2.4 m ^(6)^
*f_mod_/pulse rate*	1 GHz	100 kHz	40 MHz	10 MHz	12.5 MHz	62.5 MHz
Pulse width	-	100 ps	40 ps	150 ps	80 ps	-
*λ*	783 nm	810 nm	637 nm	650 nm	473 nm	783 nm

^(1)^ Pad-limited. Active area with SPAD, quenching circuit, and two correlators about 300 × 300 µm^2^. ^(2)^ Achieved by averaging 1000 successive measurements. Single-shot precision is 20 mm. ^(3)^ Calculated with Equation (1) in [1], by removing PDP and fill factor FF at a distance of 4 m with a pulse rate of 100 kHz. ^(4)^ 1000 successive measurements with 100 kHz pulse rate. ^(5)^ Calculated from frame rate/measurement rate. ^(6)^ Limited by unambiguous range. Can be greatly extended, e.g., with orthogonal coding. ^(7)^ Measured with prior knowledge of the scene.

## Data Availability

Data is contained within the article.
